# Spatiotemporal Molecular Analysis of Cyanobacteria Blooms Reveals *Microcystis* *-* *Aphanizomenon* Interactions

**DOI:** 10.1371/journal.pone.0074933

**Published:** 2013-09-27

**Authors:** Todd R. Miller, Lucas Beversdorf, Sheena D. Chaston, Katherine D. McMahon

**Affiliations:** 1 Department of Bacteriology, University of Wisconsin, Madison, Wisconsin, United States of America; 2 Department of Civil and Environmental Engineering, University of Wisconsin, Madison, Wisconsin, United States of America; University of New South Wales, Australia

## Abstract

Spatial and temporal variability in cyanobacterial community composition (CCC) within and between eutrophic lakes is not well-described using culture independent molecular methods. We analyzed CCC across twelve locations in four eutrophic lakes and within-lake locations in the Yahara Watershed, WI, on a weekly basis, for 5 months. Taxa were discriminated by length of MspI-digested *cpcB*/*A* intergenic spacer gene sequences and identified by comparison to a PCR-based clone library. CCC across all stations was spatially segregated by depth of sampling locations (ANOSIM R = 0.23, *p* < 0.001). Accordingly, CCC was correlated with thermal stratification, nitrate and soluble reactive phosphorus (SRP, R = 0.2-0.3). Spatial variability in CCC and temporal trends in taxa abundances were rarely correlative between sampling locations in the same lake indicating significant within lake spatiotemporal heterogeneity. Across all stations, a total of 37 bloom events were observed based on distinct increases in phycocyanin. Out of 97 taxa, a single *Microcystis* , and two different *Aphanizomenon* taxa were the dominant cyanobacteria detected during bloom events. The *Microcystis* and *Aphanizomenon* taxa rarely bloomed together and were significantly anti-correlated with each other at 9 of 12 stations with Pearson R values of -0.6 to -0.9 (*p* < 0.001). Of all environmental variables measured, nutrients, especially nitrate were significantly greater during periods of *Aphanizomenon* dominance while the nitrate+nitrite:SRP ratio was lower. This study shows significant spatial variability in CCC within and between lakes structured by depth of the sampling location. Furthermore, our study reveals specific genotypes involved in bloom formation. More in-depth characterization of these genotypes should lead to a better understanding of factors promoting bloom events in these lakes and more reliable bloom prediction models.

## Introduction

In many eutrophic lakes, cyanobacteria are responsible for massive accumulations of biomass, otherwise known as "blooms," whether formed through growth, horizontal and vertical migration, or physical forcings (e.g. wind) [1,2]. The incidence and severity of these blooms are thought to be escalating, particularly in the northern hemisphere, due to increased eutrophication of waterways and climate change [3-5]. During summer in northern temperate eutrophic lakes, the most commonly encountered genera include *Microcystis* *,* *Anabaena* , and *Aphanizomenon* , while *Phormidinium*, *Planktothrix* , *Gloeotrichia* and others occur sporadically [6,7]. In addition, invasive species from tropical/subtropical regions, including *Cylindrospermopsis* *raciborskii* have been detected recently in northern temperate eutrophic lakes, including this study [6,8]. While a natural occurrence, these blooms are exacerbated by human impacts and potentially problematic as they may lead to a decline in dissolved oxygen, produce undesirable odors and/or contain toxins harmful to humans, fish and other wildlife. For this reason, lake restoration efforts and mandates by the U.S. Environmental Protection Agency (e.g. total maximum daily loads program) are aimed at reducing the intensity and frequency of cyanobacterial blooms.

The success of freshwater cyanobacteria is attributed to a variety of intersecting circumstances—including warm water temperatures, pH buffering above neutrality, nitrogen fixation, vertical movement via gas vacuoles, carbon/phosphorus/nitrogen storage mechanisms, and colony formation that inhibits predation [9-13]—which may give them a competitive advantage over eukaryotic photoautotrophs [14]. These traits vary whether characterized by species, strain, and/or genotype [15,16]. Species interactions, including competition and synergism, may also be important for bloom development and/or toxin production since changes in specific genotypes of the same genera are observed during bloom formation and decline [17-19]. In addition, new nitrogen input from nitrogen fixing cyanobacteria may be important for growth and/or toxin production by non-nitrogen fixing cyanobacteria such as *Microcystis* [20].

Variability in cyanobacterial community composition (CCC) has been studied in lakes for decades resulting in a rich understanding of environmental factors involved in promoting their growth (reviewed by [2]). However, it is difficult to predict within any given season the timing and severity of bloom events that may form over the course of days. Furthermore, the majority of information about the ecology of cyanobacteria comes from studies relying upon culturing or microscopic identifications. Recent studies show that such techniques do not reflect the bulk of cyanobacterial diversity in lakes and thus may group functionally distinct taxa as one [21]. This is also indicated by laboratory studies, which show that strains or genotypes of the same species differ in their phenotypic responses to environmental cues (e.g. light, nutrients, colony formation) [15,16,22]. Recent field studies show shifts in genotypes of the dominant taxa of the same genera over spatial, temporal, or chemical/physical gradients [17,19,23,24]. Therefore, an analysis of CCC using culture- independent molecular methods may lead to a better understanding of taxa or genotypes responsible for bloom events and the conditions under which these occur. However, few or no studies have analyzed culture- independent molecular diversity of cyanobacteria across multiple lakes and bloom events at resolved time scales (e.g. weekly).

In this study, we investigated variability in CCC on a weekly basis over the course of thirty-seven cyanobacterial bloom events occurring at twelve sampling locations in four eutrophic lakes of the Yahara Watershed, Wisconsin, USA. We used a culture-independent molecular method to characterize CCC and asked whether variability in CCC tracked with physical and chemical lake characteristics both spatially (i.e. within and between lakes) and temporally. In addition, we compared CCC and environmental conditions between bloom events to determine the number and identity of taxa responsible for each bloom event, and the prevailing environmental conditions under which blooms occurred by these cyanobacterial taxa. In the year this study was conducted (2008), Southern Wisconsin experienced the highest precipitation on record over a 24-day period in May-June resulting in record high stream flows (500 years) and significant flooding [25].

## Materials and Methods

No specific permits were required for the described field study, nor were any specific permissions needed to sample the locations. None of the lakes sampled are privately owned, and our sampling did not involve endangered or protected species.

### Description of study stations and sampling

**Figure 1:**
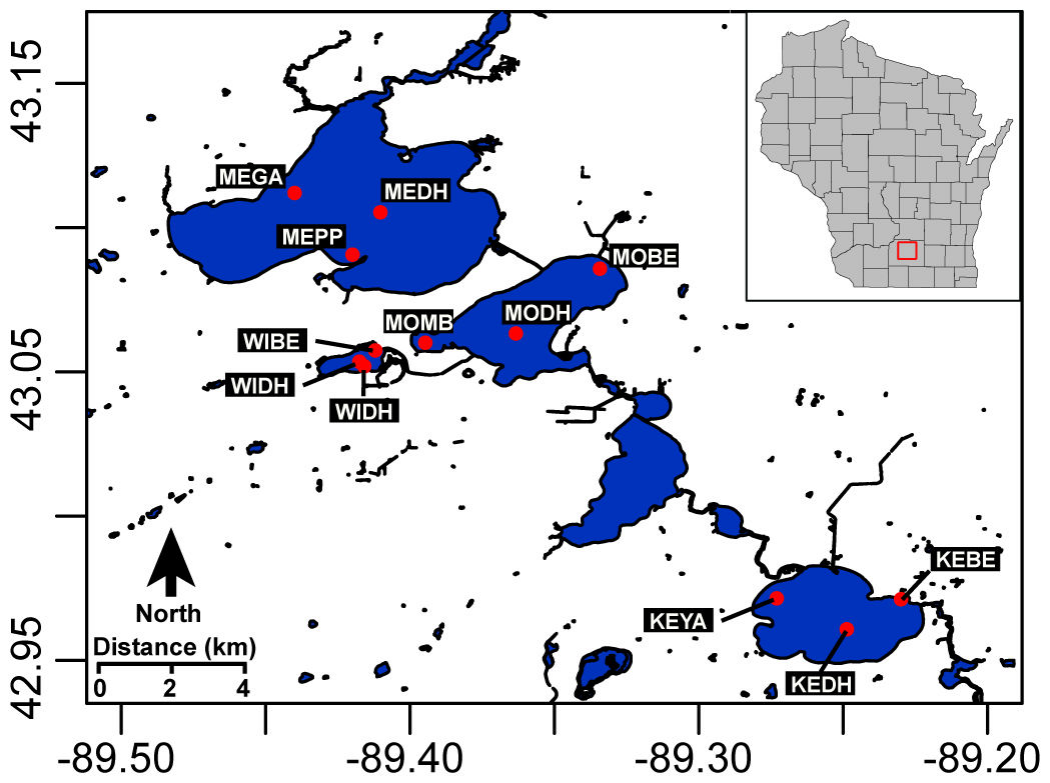
Sampling locations in lakes of the Yahara Watershed. Three locations in each of four lakes were sampled on a weekly basis late May through October.

The Yahara River connects a chain of four lakes within the Southern portion of the Yahara watershed in South Central Wisconsin, USA (Figure 1). Lake Mendota at the top of the chain is the largest, followed by Monona, Waubesa (not sampled in this study) and Kegonsa. Lake Wingra is a smaller lake situated apart from the lake chain and is fed by mainly urban runoff and a natural spring [26]. It empties into Lake Monona and is the only lake in this study that does not receive water from the Yahara River or other lakes. All of the lakes are eutrophic and have been heavily impacted by nutrients since the capital city of Madison was settled in the early 1800’s. Lake Mendota has been the subject of scientific studies for nearly a century [27]. No permits were required to sample these lakes as they are in the public domain.

Integrated samples of the water column receiving photosynthetically active radiation (photic zone depth, PZD, estimated by multiplying secchi disk depth by 1.7 [28]) were taken weekly from three locations in each lake, from late May through October 2008 using a 2" diameter PVC pipe as previously described [29]. On average, 60 samples were taken from each lake on 20 different dates. A buoy was deployed in Lake Mendota as part of the Global Lakes Ecological Observatory Network (www.gleon.org). The buoy was equipped with a phycocyanin fluorometric sensor (Cyclops 7, Turner Designs), data logger (CR-1000, Campbell Scientific) and an RS-232 modem sending data to a shore base station in near real-time. Additional samples were taken from this location when the phycocyanin fluorescent signal was high (steady increase to >2 volts) suggesting that a bloom event may be underway. Water samples were transported back to the laboratory on ice and filtered through a 47 mm, 0.2 µm pore size, polyethersulfone membrane (Supor-200, Pall Corporation). Filters were placed into 2 ml cryogenic tubes containing 1 g of autoclaved 2 mm glass beads and stored at -80 °C for DNA extraction. A 50 ml aliquot of sample water was amended with 0.05 ml of concentrated HCl and stored at 4 °C for measurement of total phosphorus (TP) and 50 ml of water was filtered through a 0.7 µm glass fiber filter (GF/F, Whatman) and stored at -20 °C for measurement of soluble reactive phosphorus (SRP), nitrate, and nitrite.

### Analytical procedures

SRP was measured in filtered samples by the ascorbic acid method 4500 PE [30]. TP was measured as SRP in unfiltered, acidified samples following persulfate digestion in an autoclave for 1 hour at 120°C [30]. Nitrate and nitrite were measured in filtered samples by HPLC as previously described [31]. Phycocyanin was extracted from filters by bead beating in 20 mM sodium acetate (pH 5.5). The extract was centrifuged to remove cell debris and phycocyanin was measured spectrophotometrically at 620 nm with correction at 650 nm [32].

### DNA extraction and automated phycocyanin intergenic spacer analysis (APISA)

CCC was determined based on sizing of MspI-digested PC-IGS gene fragments on an automated sequencer, which we call automated phycocyanin intergenic spacer analysis or APISA. It is a variation on terminal restriction fragment length polymorphism analysis, except that the size of fragments is based on the MspI site as well as length of the intergenic spacer. Sizing on an automated sequencer allows for discriminating fragments that differ by 1-4 bp and an estimate of relative abundance. Briefly, DNA was extracted directly from filters using a xanthogenate-phenol-chloroform protocol previously described [6]. The *cpcB/A* genes plus intergenic spacer region were amplified from total community DNA using primers previously published [33]. Each 50 µl PCR consisted of 2 µl template DNA, 250 µM dNTP’s, 0.4 µM of each primer, 3 mM MgCl_2_, 0.25 mg/ml BSA, 1 unit of GoTaq Polymerase (Promega), and 5 µl of 10X buffer supplied with Taq. The thermocycler protocol was as previously described [33]. The PCR products were precipitated and desalted with absolute ethanol and ammonium acetate, respectively, and subjected to digestion with MspI restriction endonuclease (Promega) for 2 hours at 37 °C and the reaction stopped at 65°C for 10 min. The digested PCR products were desalted, precipitated, and resuspended in 20 µl of ddH_2_O. A 3 µl aliquot was mixed with 10 µl of formamide and 0.4 µl of a ROX labeled (Carboxy-X-rhodamine) custom size standard (Bioventures) containing DNA fragments ranging from 30-1250 bp. The APISA DNA fragments were sized by capillary electrophoresis using an ABI 3700 sequencer as previously described [34].

### Analysis of APISA data

Electropherograms were aligned and analyzed using Genemarker® v1.75 as previously described [35]. Peak size of unknowns was determined based on mobility of size standards using the local southern method implemented within Genemarker®. Peaks were binned into operational taxonomic units (OTUs) by visually inspecting a trace overlay of all samples. Individual runs were manually checked to ensure correct calling of peaks. An arbitrary cutoff of 100 relative fluorescent units (RFUs) was used to distinguish signal from noise and only runs generating maximum peak heights of at least 3,000 RFUs were accepted. The absolute height of peaks was divided by the sum of peak heights within each run to calculate relative peak height, which was used as an estimate of relative abundance [34].

An attempt was made to assign OTU’s to genera based on comparison to a library of PC-IGS sequences. We previously provided a sequence alignment and phylogenetic analysis of 271 PC-IGS sequences recovered from the same samples as those analyzed in this study [6]. An additional 276 reference PC-IGS sequences from Genbank were included in this alignment in order to assign sequences to genus. Sequences were aligned in the ARB software environment [36] as previously described [6] and classified to genus based on their occurrence in a maximum likelihood tree relative to PC-IGS from well-characterized strains. The TRF Cut tool [37] was implemented within ARB to digest PC-IGS sequences *in silico* using the MspI restriction site. A list of terminal fragment sizes with their corresponding genus classification was then exported from ARB and used as a custom database in the Phylogenetic Assignment Tool (PAT) [38], which was used to assign a cyanobacterial genus to the OTU’s observed in our samples. For PAT analysis a size tolerance of 2 bp and 4 bp was selected for bins ranging in size from 100-250 bp, and >250 bp, respectively, based on the observation that peak width increases with elution time from the sequencer [38].

### Identification of bloom events

For this study we considered a cyanobacterial bloom to be any accumulation of phycocyanin in the photic zone (i.e. the area of the water column sampled), mathematically defined as an increase in phycocyanin 1.5X above a baseline. Each point along the baseline was calculated as a simple moving average (SMA) using all previous observations, except those identified as occurring within a bloom. The SMA is defined as:

$$SMAn=\frac{S1+S2...Sn}{N}$$

Where *S*_*N*_ does not include bloom samples. Hence, the first point on the baseline is equal to the first observed phycocyanin concentration and all subsequent points are calculated as the SMA of all previously observed phycocyanin concentrations that were below 1.5X of the baseline. Excluding bloom samples from the SMA calculation prevents stepwise increases in the baseline after each bloom event, which otherwise would underestimate the number of bloom events occurring later in the year.

### Statistics

All chemical and physical data were log transformed prior to all analyses. Correspondence (CA) and canonical correspondence analyses (CCA) were conducted in CANOCO v5.4 [39]. A matrix of relative abundances of each taxa by sample date was imported into CANOCO along with a matrix containing log- transformed chemical and physical data (i.e. environmental data) including TP, SRP, nitrate, nitrite, PZD, photic zone water temperature and DO (i.e. average water temperature and DO over the integrated depths sampled). In addition, the degree to which the water column was stratified at each location was estimated by the difference in water temperature at the surface minus temperature at the lake bottom (**Δ**T). For the CA and CCA’s, a response model was chosen using indirect or direct gradient analyses, respectively. Biplot scaling was selected with focusing on inter-sample distances. The OTU data was not further transformed and only one outlier was removed. An Analysis of Similarity (ANOSIM) was conducted in Primer v5.0 using a Bray-Curtis dissimilarity matrix. All descriptive statistics, Pearson R correlations, and Mann-Whitney tests were calculated in the R statistical package.

## Results

### Trends in chemical and physical lake variables

Trends in water temperature were similar across all stations and ranged from 11.5-30 °C over the sampling period. PZD became shallow at all stations early in June and remained shallow averaging 2.7 +/- 2.0 m for the rest of the sampling period (Figure S1). We defined shallow stations as <3.7 m and all others as deep locations, which were all greater than 10 m. At shallow locations PZD extended through nearly the entire water column on most sampling days (Figure S1). Thermal stratification (ΔT) dissipated at all stations in June, due to a larger storm event, and again in October, due to lake mixing in autumn, but remained weak at shallow stations for the entire sampling period (Table 1, Figure S1).

**Table 1:** Mean (+/- Standard Deviation) of physical and chemical lake characteristics.

**Site**	**Lake**	**Depth(m)**	**T°C**	**^a^ΔT**	**^b^PZD**	**^c^Nitrate(µg/L)**	**^c^Nitrite(µg/L)**	**TP(µg/L)**	**SRP(µg/L)**
MEDH	Mendota	25.7	21.7 (3.8)	9.7 (3.9)	3.7 (2.7)	190 (126)	69 (46)	49 (38)	33 (45)
MEGA	Mendota	18.1	20.1 (3.5)	7.8 (4.0)	6.0 (3.8)	243 (208)	56 (75)	50 (32)	63 (54)
MEPP	Mendota	2.3	21.3 (2.1)	0.7 (0.5)	3.5 (0.9)*	278 (208)	94 (106)	48 (35)	34 (41)
MODH	Monona	23.1	22.8 (3.6)	7.3 (3.5)	3.1 (2.1)	82 (149)	161 (253)	35 (28)	23 (19)
MOBE	Monona	3.6	23.3 (3.0)	0.9 (1.3)	2.3 (0.6)	67 (62)	109 (227)	50 (41)	29 (29)
MOMB	Monona	1.8	22.4 (4.9)	0.9 (0.7)	1.3 (0.7)	56 (54)	64 (111)	61 (33)	22 (25)
KEDH	Kegonsa	9.6	22.8 (3.9)	1.4 (1.4)	2.7 (1.8)	87 (33)	65 (117)	94 (45)	47 (42)
KEBE	Kegonsa	1.2	22.2 (4.3)	0.4 (0.5)	1.9 (0.6)*	89 (170)	70 (118)	91 (31)	36 (41)
KEYA	Kegonsa	2.1	23.5 (3.6)	0.7 (0.5)	2.7 (0.8)*	41 (37)	43 (96)	85 (34)	40 (44)
WIDH	Wingra	3.7	24.8 (3.8)	1.1 (1.0)	1.9 (0.6)	BDL	BDL	20 (10)	14 (7)
WIAR	Wingra	2.8	23.6 (4.3)	0.6 (0.6)	1.8 (0.2)	BDL	BDL	23 (13)	13 (8)
WIBE	Wingra	2.3	24.5 (3.6)	0.7 (0.6)	1.7 (0.6)	BDL	BDL	23 (11)	13 (5)

a ΔT is the difference in water temperature at the bottom and top of the lake at each site.

^b^ PZD was estimated by multiplying secchi disk depth by 1.7. As such, average PZD calculated at some locations (*) extends beyond the maximum depth.

c Nitrate and nitrite were below detection limits (BDL) in Lake Wingra on the majority of sample dates.

At all locations, dissolved nutrients were highest in June, well after spring mixing, possibly due to heavy rain events, and again in October due to lake mixing in autumn (Figure S1). Nutrients also followed similar trends across all lake locations with two exceptions. Nitrate and nitrite were below detection limits (0.01 mg/L) at all sampling locations in Lake Wingra for nearly the entire sampling period. In both Lake Kegonsa and Wingra, TP followed an opposite trend compared to the other lakes with highest concentrations in August and/or September, depending on lake location (Figure S1). Increases in TP in these lakes were presumably due to sediment resuspension in these shallower, polymictic lakes. At the north end of the lake chain, average concentrations of nitrate in Lake Mendota were 2-7 times higher than all other lakes (Table 1) while SRP was similar across Lakes Mendota, Monona, and Kegonsa, but significantly lower in Lake Wingra. The SRP may have been lower in Lake Wingra because this lake is not subject to agricultural runoff and a larger percentage of the lake supports macrophyte growth of Eurasian milfoil ( *Myriophyllum* *spicatum* ).

### Identification of cyanobacteria

**Figure 2:**
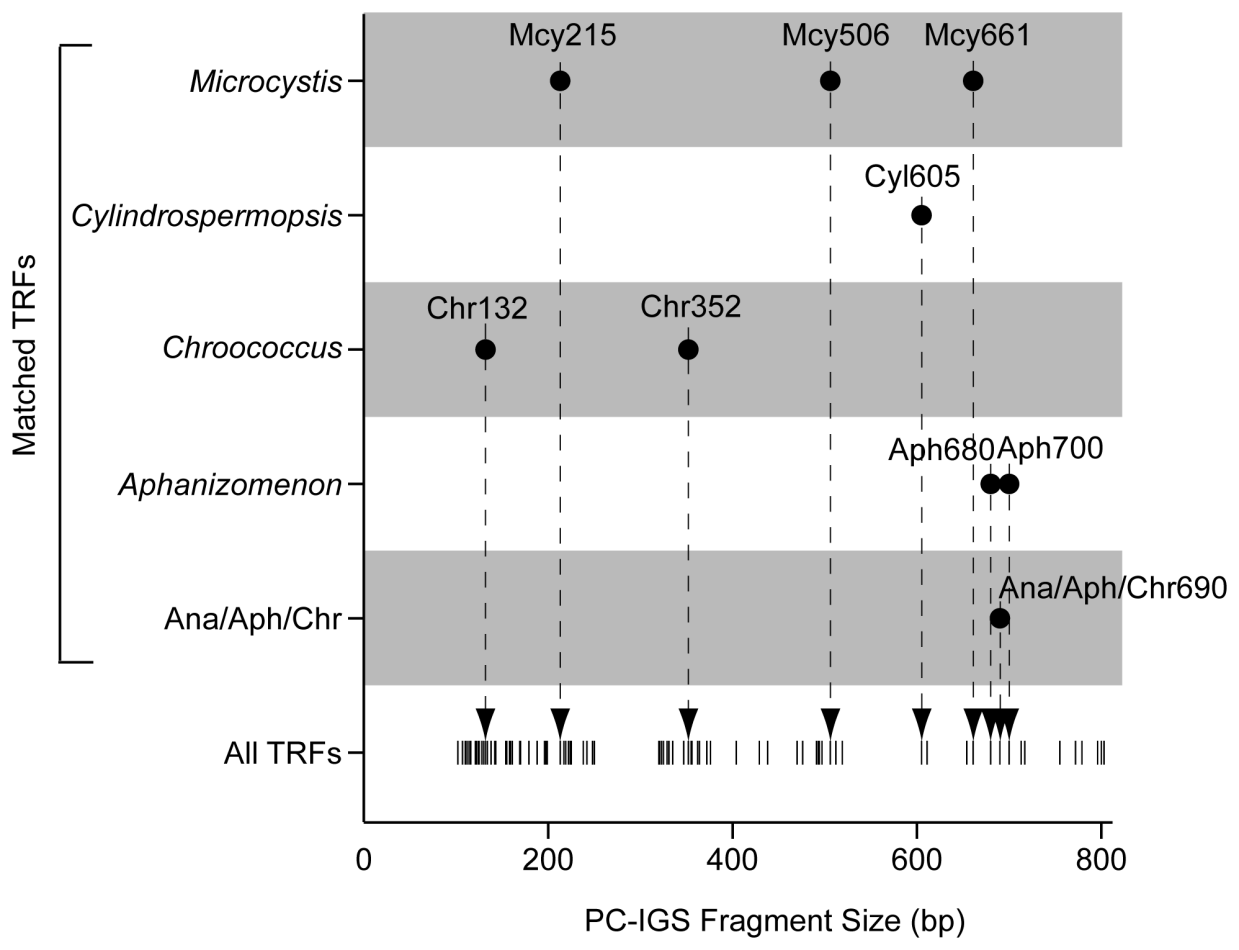
Identification of cyanobacteria represented by APISA OTU’s. Nine of the 94 unique PC-IGS terminal fragments (TRFs) detected among 197 sample could be matched by size to an *in* *silico* MspI digestion of PC-IGS sequences previously retrieved from the same samples. These were the most abundant TRFs detected representing 87.1 ± 13.5% of the total peak heights in any given sample indicating that these are the most abundant taxa in our samples.

To identify cyanobacteria, the APISA OTUs were matched to phylogenetically characterized PC-IGS sequences digested with MspI *in silico*. There were 94 total OTUs detected, and nine of these could be matched to the PC-IGS sequences digested *in silico* with MspI (Figure 2). The nine OTUs identified were from *Microcystis* , *Aphanizomenon* , *Chroococcus* , and *Cylindrospermopsis* taxa, which agrees with our previous identification of cyanobacteria in these lakes based on PCR-based clone libraries [6]. One APISA OTU (i.e. AnaAphChr690) represented sequences from three different genera ( *Anabaena* , *Aphanizomenon* , and *Chroococcus* ). Therefore, the identity of this OTU is ambiguous among these three genera and was not included when summing relative abundances of genera.

The nine OTUs that could be identified accounted for on average 87 ± 14% of the total peak heights detected in any given sample. None of the unidentified OTUs were significantly abundant in any one sample and were rarely detected in more than three samples. However, the sum of relative abundances of all unidentified OTUs was on average the second highest at each sampling station. This suggests that a diverse community of less abundant cyanobacterial taxa exists in these lakes that are not represented by known PC-IGS sequences. The *Microcystis* (Mcy) OTU designated Mcy215 was on average the most abundant in the entire dataset followed by unidentified taxa> AnaAphChr690> *Aphanizomenon* (Aph) 680> Mcy661> Mcy506> Aph700> *Chroococcus* (Chr) 352> Chr132, and *Cylindrospermopsis* (Cyl) 605. Collectively, the *Microcystis* taxa made up, on average, about half of the relative abundance at each sampling location (51 +/- 9%).

### Spatial and temporal variability in cyanobacterial community composition

**Figure 3:**
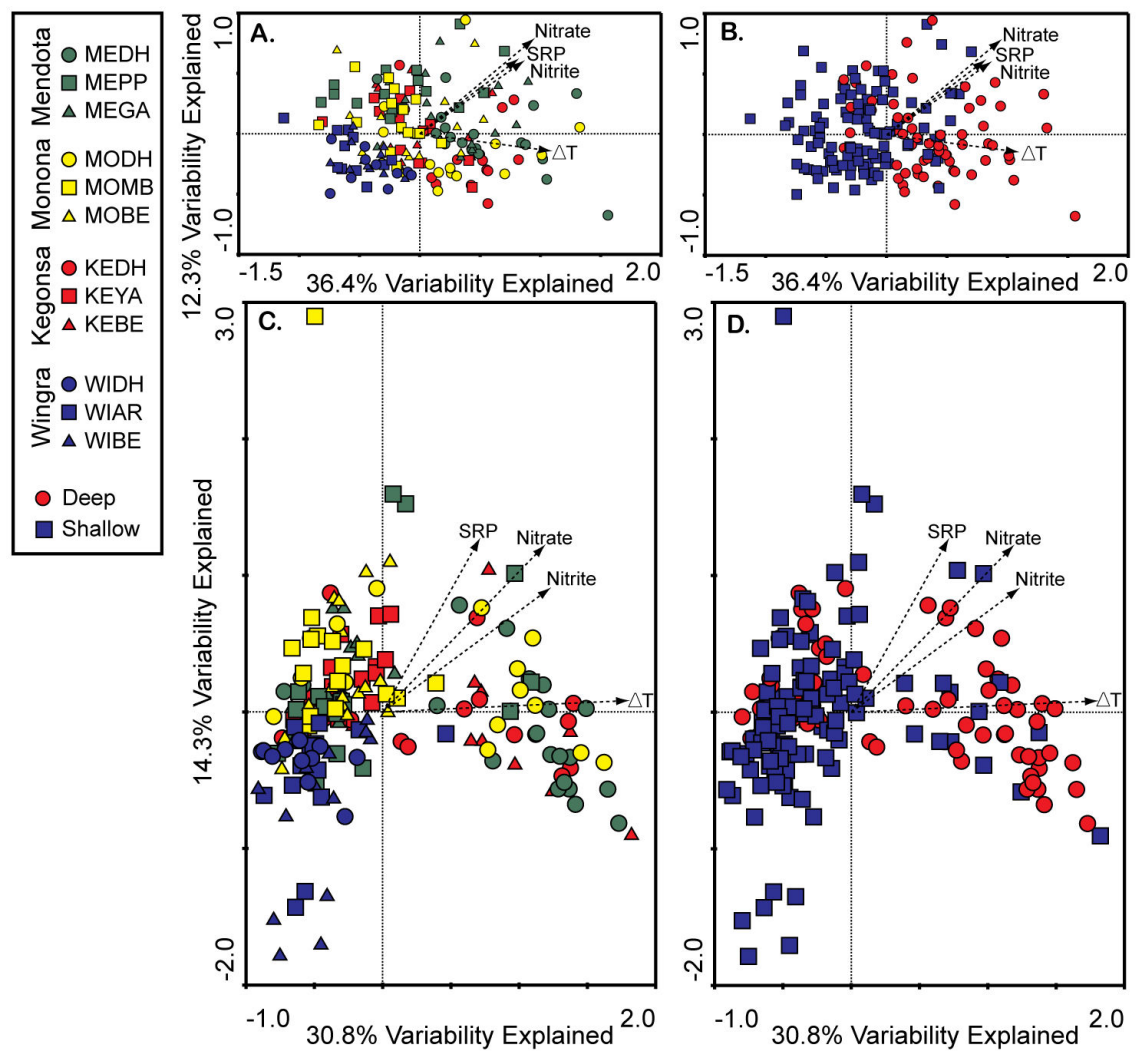
Spatial variability in cyanobacterial community composition based on correspondence analysis of all samples (n= 197), constrained (A and B) or not constrained (C and D) by environmental variables. Samples are coded by sampling site (A and C) or depth of the water column at each sampling site (B and D). Spatial variability in CCC is most strongly segregated between shallow (<3.7 m) and deep- water sites (> 10 m). Stratification (difference in water temperature from surface to lake bottom, ∆T) correlates best with spatial variability in CCC along the first axis.

A total of 228 samples were collected across the four lakes and twelve stations (17-26 samples per station). Of these, 197 were analyzed by APISA allowing for an analysis of both temporal and spatial variability in CCC. Less samples were analyzed in Lake Wingra due to lack of PCR amplification of the PC-IGS, which is consistent with lower levels of phycocyanin in this lake (Figure S1). Correspondence analysis was undertaken to explore relationships between CCC and environmental variables between sampling stations (Figure 3). The analysis was either unconstrained (Figure 3A and B) or constrained (Figure 3C and D) by environmental variables, the latter assumes all variation in CCC is accounted for by measured environmental variables. Overall, CCC was segregated between deep and shallow (<3.7 m) stations (ANOSIM R=0.23 ,p < 0.001) in both types of ordinations along the first axis and accounted for 36.4% or 30.8% of the variability in the dataset using constrained or unconstrained analyses, respectively (left to right, Figure 2B and 2D). Average ΔT was higher at deep-water sampling locations (Table 1) and variability in CCC along the first axis correlates best with ΔT (Table 2).

**Table 2:** Spatial trends in cyanobacterial community composition with environmental variables based on correspondence analysis.

**Variable**	**Unconstrained (R=)**		**Constrained (R=)**	
	**Axis 1**	**Axis 2**	**Axis 1**	**Axis 2**
**TP**	0.15	**0.27**	0.24	0.14
**SRP**	0.15	**0.34**	0.28	**0.29**
**Nitrate**	**0.25**	**0.33**	**0.35**	**0.39**
**Nitrite**	**0.26**	**0.25**	**0.32**	**0.29**
**ΔT**	**0.38**	0.02	**0.40**	-0.07
**Temp**	0.07	-0.11	0.06	**-0.35**
**DO**	**0.25**	0.14	**0.32**	-0.09
**PZD**	0.2	0.19	0.28	0.27

Of all taxa, the average relative abundance of the *Microcystis* OTU designated Mcy215 was significantly higher at shallow water stations and was twice as high in the shallowest lake, Lake Wingra than in all other lakes (Figure S3). The same was not true for the other *Microcystis* taxa, which followed an opposite trend with lowest abundance in Lake Wingra. This accounted for the segregation in CCC between deep and shallow locations.

Shallow locations were also segregated from lower left to the upper left quadrants in the ordination (Figure 3A and 3C). This split was primarily between Lake Wingra samples and all other shallow locations (ANOSIM R= 0.3-0.7, *p* < 0.002). Variability in CCC along this axis correlated best with nitrate, SRP, and nitrite (Table 2) due to the fact that nitrate and nitrite were below detection limits in Lake Wingra and average SRP was 2-3 times lower in Lake Wingra than in other locations (Table 1). There were some significant but modest differences (ANOSIM R = 0.1-0.4, *p* < 0.05) in CCC between several locations in the same lake indicating within lake heterogeneity. Many of these significant differences were between deep and shallow locations (Table S1).

We asked whether relative abundance of individual taxa followed the same trend at different sampling locations in the same lake. The unidentified taxa were not included in this analysis since individual unidentified taxa were only detected on a few sampling days at each location. There was a striking difference between Lake Mendota and all other lakes. In Lake Mendota, the trends in relative abundance for any given cyanobacterium, except for one (i.e. Chr132), were similar at all three stations with Pearson R values of 0.6 or greater (*p* < 0.05). In other words, the same OTU followed the same trend at all three stations. In contrast, with just a few exceptions, individual cyanobacterial taxa in the other three lakes (Monona, Kegonsa, and Wingra) did not follow the same trend between locations in the same lake at a significance cutoff of *p* < 0.05. For example, in Lake Kegonsa on Sept. 5^th^, Aph680 was responsible for a bloom at the KEDH location, but was not detected on the same date, 1.9 km away in the same lake at the KEBE location. Only a few taxa followed similar trends in these three lakes. For example, *Cylindrospermopsis* in Lake Monona (R = 0.6, *p* = 0.05), Aph700 in Lake Kegonsa (R = 0.9, *p* < 0.001), and two *Aphanizomenon* taxa and Chr352 in Lake Wingra. Overall, these data indicate that with the exception of Lake Mendota, most cyanobacterial taxa follow contrasting trends in relative abundance between locations in the same lake.

In 7 out of 9 locations, temporal variability in CCC correlated best with either PZD (R = 0.60-0.66) or SRP (R = 0.60-0.85) along the first axis accounting for 21.2-34.9% of the variability (Table 3). The second axis was most strongly correlated with either ΔT, temp, or SRP accounting for 18.9-23.8% of the variability. Variability in CCC was not strongly correlated with time (i.e. days since sampling began) and a temporal or seasonal trajectory in CCC was not evident at most locations. The data suggest that variability in CCC through time at each location was most strongly influenced by changes in PZD and SRP.

**Table 3:** Temporal Trends in Cyanobacterial Community Composition with Environmental Variables Based on Canonical Correspondence Analysis.

**Site**	**FirstAxis(Variable,R^a^**)		**%Var**	**SecondAxis(Variable,R**)		**%Var**
MEDH	PZD, 0.60		31.7	SRP, 0.54		20.2
MEPP	SRP, 0.85	DO, 0.59	34.9	PZD, -0.61		18.9
MEGA	SRP, -0.60		30.5	SRP, 0.58	DO, 0.55	21.0
MODH	PZD, 0.60		31.7	DO, 0.53		20.2
MOMB	NO3, -0.54		21.2	Temp, 0.61	TP, -0.51	19.3
MOBE	Temp, -0.56		25.7	None		23.8
KEDH	PZD, 0.60		31.7	SRP, 0.54		20.2
KEYA	SRP, -0.60		29.5	SRP, 0.58	DO, 0.55	19.6
KEBE	PZD, -0.66		28.6	Temp, -0.65	SRP, -0.53	20.9
WIDH^b^	DO, -0.73	SRP, 0.61	52.8	PZD, -0.57		16.0
WIAR^b^	None		35.0	None		23.1
WIBE^b^	Temp, -0.66	PZD, 0.58	36.1	None		22.2

a Variables that correlate with temporal changes in CCC having R>0.5 (or R< -0.5) are shown. SRP= soluble reactive phosphorus, PZD= photosynthetically active radiation, Temp= average water temperature across the depths sampled, NO3= nitrate.

b The analysis of Lake Wingra samples did not include nitrate since nitrate was below detection limits in the majority of samples from all stations in this lake.

### Timing of bloom events

**Figure 4:**
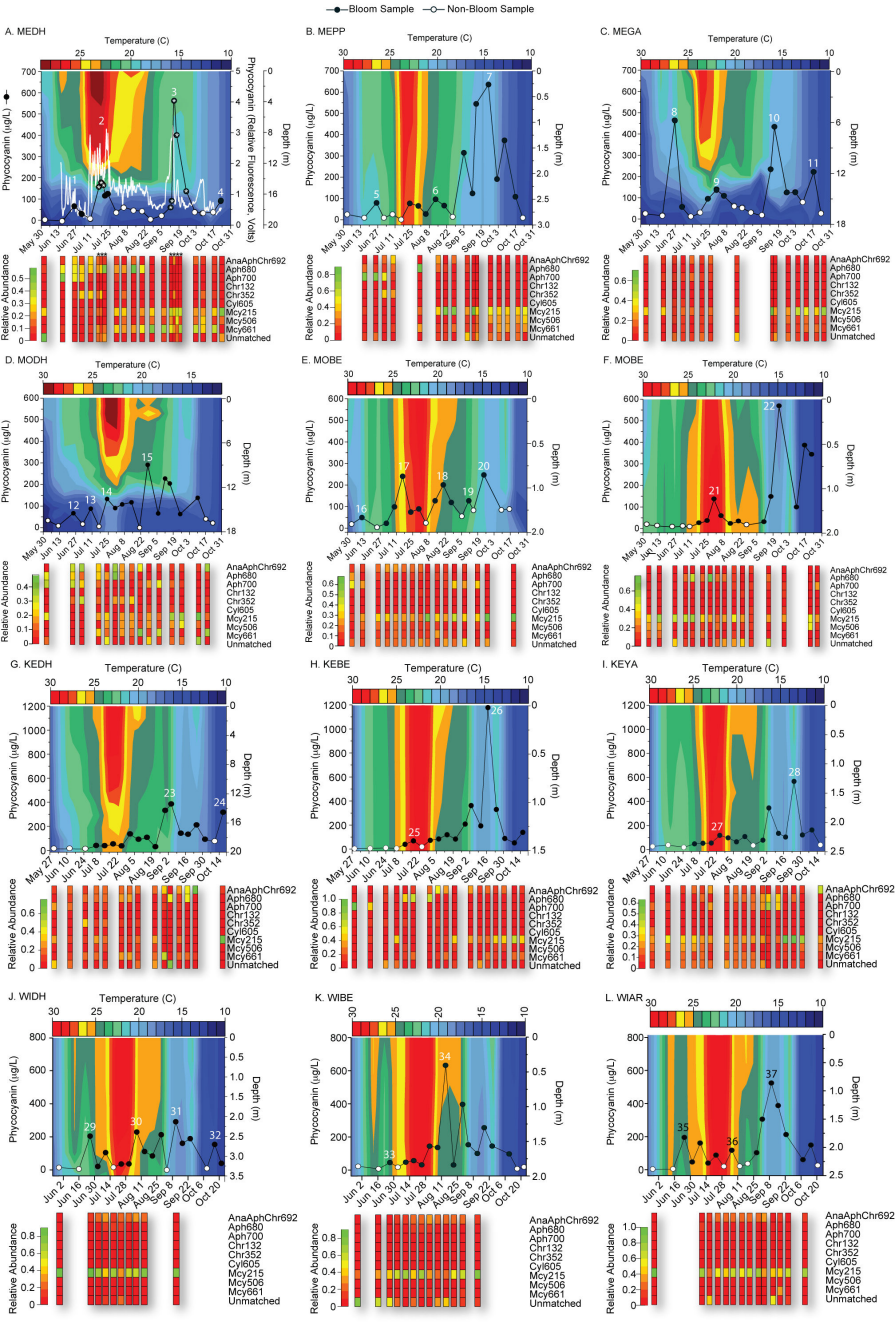
Trends in phycocyanin concentration (line graphs) and water temperature by depth (contour maps) compared to relative abundance of cyanobacterial taxa (bottom heat maps). Closed circles represent dates when a bloom was occurring. At the MEDH location an in- situ fluorometer attached to a monitoring buoy measured phycocyanin fluorescence once per minute (white line graph).

Significant increases in phycocyanin were observed in all lakes during the sampling period resulting in blue-green colored scums on the water surface (Figure S2), primarily during late summer and early Autumn. Phycocyanin levels varied by up to two orders of magnitude between weekly samplings (Figure 4). At station MEDH, where adaptive sampling was practiced, phycocyanin concentrations were observed to increase 100-fold, from 62 μg/L to nearly 600 μg/L phycocyanin within a 24-hour period (i.e. Sept 12 – Sept. 13) indicating significant temporal variability in cyanobacterial biomass at sub-weekly time-scales in a given location. The coefficient of variation for phycocyanin was 1.19 with an average and standard deviation of 113 +/- 135 μg/L.

A bloom event was defined as sustained levels of phycocyanin 1.5X above the simple running average of phycocyanin concentrations not including bloom samples. This definition generally agreed with qualitative observations of water quality during sampling. However, there were several events where this definition grouped multiple, often, lesser phycocyanin increases into one bloom event. These occurred in Lakes Kegonsa and Wingra (Figure 4). Therefore, our bloom definition is a conservative estimate of the number of bloom events that occurred in each location, which is consistent with the significant temporal variability observed from adaptive sampling in Lake Mendota.

The average bloom duration was 29 +/- 25 days. Some blooms lasted for as little as 4 days while others slowly accumulated and then declined over the entire sampling period resulting in a single event lasting > 3 months (Table 4 and Figure 4). Since sampling was on a weekly basis it is possible that some events were missed, or that multiple blooms were grouped into one. Trends in phycocyanin concentration between any two sampling stations, (i.e. within or between lakes) were not significantly correlated with each other at a cutoff of *p* < 0.05 indicating significant within lake bloom heterogeneity. However, the start of the cyanobacterial bloom season at all stations occurred simultaneously, within one to two weeks of each other in mid-June to early-July. This was coincident with increasing water temperature, declining dissolved nutrient concentrations, and an increase in ΔT (Figure S1).

**Table 4:** Dominant taxa during mild, moderate, or severe cyanobacterial blooms at each sampling location.

**Site**	**Bloom#**	**^a^Date**	**Days**	**^b^Level**	**^c^Dominant Taxa**
**MEDH**	1	Jun 26	13	Mild	Aph680, Aph700, Chr352
	2	Jul 18	14	Moderate	Aph680, Chr352
	3	Sep 15	19	Severe	Mcy215, Mcy506
	4	Oct 23	ND	Mild	Mcy215, Mcy506
**MEPP**	5	Jun 24	8	Mild	Aph700
	6	Aug 13	35	Mild	AnaAphChr690, Mcy215
	7	Sep 24	48	Severe	Mcy215, Mcy506
**MEGA**	8	Jun 26	20	Severe	None
	9	Jul 30	13	Mild	ND
	10	Sep 15	26	Moderate	Mcy215
	11	Oct 17	6	Moderate	Mcy215, Mcy661
**MODH**	12	Jun 24	8	Mild	AnaAphChr690, Aph680, Aph700
	13	Jul 9	7	Mild	ND
	14	Jul 23	21	Mild	Mcy215
	15	Aug 27	50	Moderate	None
**MOBE**	16	Jun 11	13	Mild	Aph700, Mcy215
	17	Jul 16	34	Moderate	Mcy215
	18	Aug 20	23	Moderate	Mcy215
	19	Sep 11	4	Mild	ND
	20	Sep 24	15	Moderate	Mcy215
**MOMB**	21	Jul 30	41	Mild	None
	22	Sep 24	41	High	Mcy215
**KEDH**	23	Sep 3	98	Moderate	Aph680
	24	Oct 16	ND	Moderate	Mcy215
**KEBE**	25	Jun 24	9	Mild	None
	26	Sep 17	98	Severe	Mcy215, Mcy661
**KEYA**	27	Jul 24	42	Mild	ND
	28	Sep 3	41	Moderate	Aph700, Aph680
**WIDH**	29	Jun 26	21	Moderate	Mcy215
	30	Aug 7	41	Moderate	AnaAphChr690, Mcy215
	31	Sep 11	28	Moderate	Mcy215
	32	Oct 16	>6	Moderate	ND
**WIBE**	33	Jun 26	7	Mild	Mcy215
	34	Aug 14	91	Severe	Mcy215
**WIAR**	35	Jun 26	28	Moderate	ND
	36	Aug 7	7	Mild	AnaAphChr690, Mcy215
	37	Sep 11	54	Severe	Mcy215

a The peak of the bloom is defined as the date when maximum phycocyanin concentrations occurred.

c The severity of bloom events was defined by the concentration of phycocyanin (µg/L) at the peak of the bloom event. Mild = <150, Moderate = 150-500, Severe = >500.

b Dominant taxa detected at the peak of the bloom event. ND = Not determined, None = no dominant taxa representing >20% relative abundance.

### Dominant taxa during blooms

*Microcystis* and *Aphanizomenon* dominated (i.e. ≥ 20% relative abundance) most bloom events (Figure 4, Table 4). While there was significant variability in phycocyanin over time the relative abundance of individual taxa did not always vary with blooms. The heat maps in Figure 4 show that Mcy215 was consistently detected at most locations and on most sampling days regardless of whether a bloom was occurring. The relative abundance of the other two *Microcystis* taxa grew significantly on only a few days in primarily the deepest locations (e.g. MEDH and MODH), but their peak relative abundances did not coincide with most bloom events (Table 4). Of the 31 bloom events analyzed by APISA, twelve were dominated by Mcy215 alone, while 9 of the 31 events were co-dominated by Mcy215 and other taxa. While no single cyanobacterium was responsible for all bloom events, Mcy215 was involved in the majority of them (i.e. 68%) and all of these were severe (i.e. phycocyanin > 500 µg/L). Of the 21 *Microcystis* - dominated bloom events all but seven occurred in August and September, corresponding to periods when SRP and nitrate were at their lowest and thermal stratification was strong.

The *Aphanizomenon* taxa dominated two bloom events alone (i.e. Aph680 or Aph700) and five together and/or with other taxa. Nearly all *Aphanizomenon* -dominated bloom events (5 out of 7) occurred early in the sampling period (i.e. June and July) and/or occurred prior to the first *Microcystis* bloom (Table 4). These five events occurred at five different stations, all early in summer (1^st^ or 2^nd^ week of June). Of all stations, only two had a *Microcystis* dominated bloom early in summer, but these occurred at the end of June and both stations were in Wingra where *Aphanizomenon* was generally absent. Seven stations did not have an *Aphnizomenon*- dominated bloom early in summer. In a few blooms, no dominant taxa were present at the peak of the bloom (i.e. none with > 20% relative abundance) indicating multiple taxa contributed to the bloom or the dominant organism could not be detected by our methods.

## Relative Abundance of *Aphanizomenon* and *Microcystis* Are Anti-Correlated

**Figure 5:**
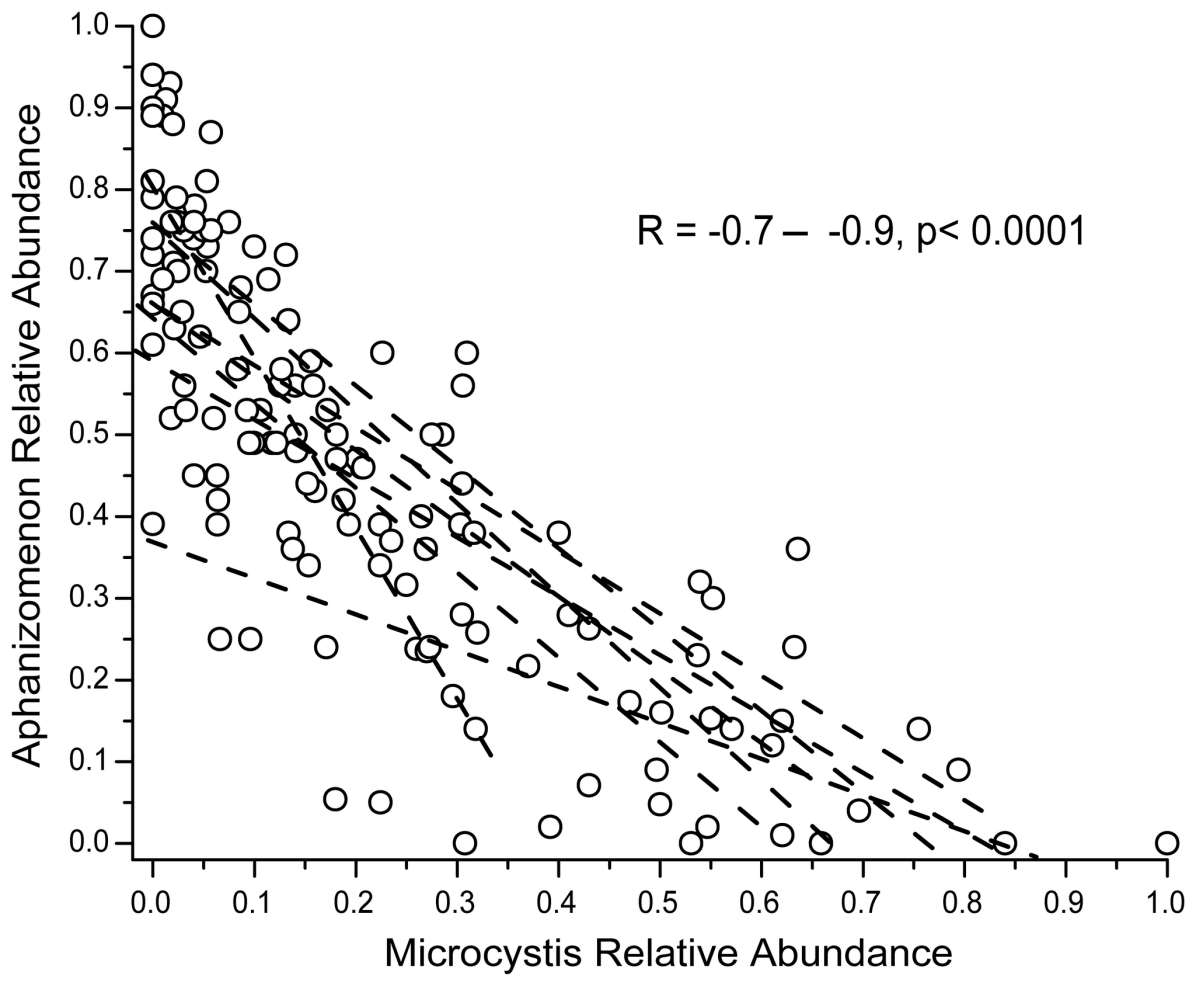
Significant negative linear relationships between *Microcystis* and *Aphanizomenon* relative abundance at nine of twelve sampling sites. The linear fit of the relationship at each of the nine sites is given by dotted lines.

While *Microcystis* and *Aphanizomenon* were the dominant taxa during most bloom events, they rarely bloomed together and only co-dominated the same bloom event on one occasion (Table 4, bloom# 16). These two genera were nearly mutually exclusive across all lakes. The sum of relative abundances of *Aphanizomenon* and *Microcystis* taxa were significantly (*p* < 0.001) anti-correlated with each other at nine of twelve stations with Pearson R values of -0.7--0.9 (Figure 5). The community cycled between *Aphanizomenon* and *Microcystis* dominance on multiple occasions at each of these nine stations and *Microcystis* was always the dominant taxa at the end of the sampling period in late October (Figure S1). No other genera (e.g. *Chroococcus* ) or taxa (e.g. AnaAphChr690) were significantly anti-correlated with either *Aphanizomenon* or *Microcystis* .

Linear trends in the dominant taxa during blooms including two *Aphanizomenon* taxa, Mcy215 and the sum of *Microcystis* or *Aphanizomenon* taxa were significantly correlated with nutrients and/or physical variables at 9 of 12 stations (Table S2). The *Microcystis* and *Aphanizomeon* taxa showed opposing correlations to the same variable at all three stations in Mendota, two in Monona and one in Kegonsa, suggesting that these genera show contrasting responses to environmental conditions. For example, at these stations *Aphanizomenon* showed strong positive correlations with SRP and/or ΔT while *Microcystis* showed negative correlations to these same variables.

**Figure 6:**
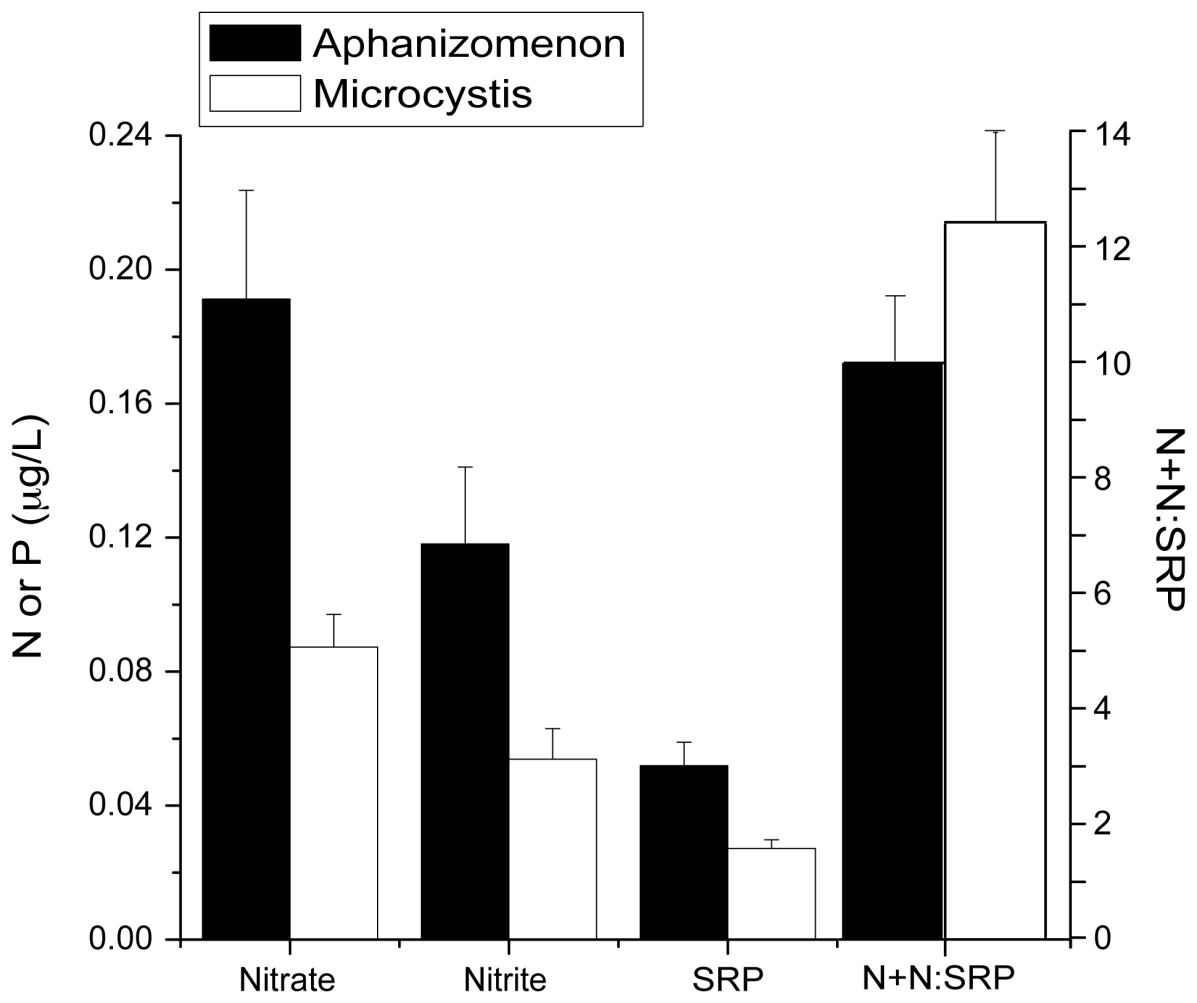
Average concentrations of dissolved nutrients during periods of *Aphanizomenon* and *Microcystis* abundance. All except N+N:SRP are significant differences (P<0.01).

Average dissolved nutrient concentrations, including nitrate, nitrite, and SRP were significantly different (*p* < 3.6 x 10^-5^) between periods of *Microcystis* and *Aphanizomenon* dominance (Figure 6). Among other variables, PAR > DO > ΔT were also important with decreasing significance (*p* > 0.01). The nitrate plus nitrite (N+N) to SRP ratio (N+N:SRP) also differed between *Microcystis* and *Aphanizomenon* abundance (i.e. lower during *Aphanizomenon* abundance), but was not significant at all stations. Together, these data suggest that *Aphanizomenon* was most often dominant during periods when nutrient concentrations were relatively high compared to periods of *Microcystis* dominance, but the ratio of available inorganic N relative to P was low, possibly favoring this nitrogen fixing cyanobacterium. At two of the three stations where *Microcystis* and *Aphanizomenon* were not anti-correlated (i.e. WIAR, and WIBE), *Aphanizomenon* relative abundance was negligible while in the third station (i.e. MOMB), *Aphanizomenon* and *Microcystis* were significantly anti-correlated with the exception of June samples.

## Discussion

In this study, we attempted to capture both spatial and temporal variability of CCC in multiple eutrophic lakes and stations using a culture-independent molecular method. We discovered that most bloom events across all lakes and stations were dominated by just three of the more than 90 taxa or genotypes identified (e.g. Aph680, Aph700, and Mcy215). Although other taxa were involved in some bloom events, at least one of these three taxa were involved in all of them. Furthermore, we found that not all *Microcystis* taxa were equal with respect to bloom formation, and that only one of the three *Microcystis* taxon identified (Mcy215) was responsible for most cyanobacterial bloom events across all stations.

With respect to accumulations in cyanobacterial biomass, our observations were consistent with previous work [40]. Blooms formed when water temperatures were above 20°C, stratification (∆T) was increasing, and dissolved nutrients were either declining or at a minimum. However, the molecular analyses showed that most of the first blooms involved the two *Aphanizomenon* taxa and occurred within 1-2 weeks of one another at all stations, while most of the subsequent blooms were dominated specifically by *Microcystis* , Mcy215. Additionally, the *Microcystis* blooms were not concurrent across stations. This suggests that *Aphanizomenon* responds to the change in season while *Microcystis* benefits from a suite of intersecting station- specific conditions. We found that seasonal trends in water column stratification predict *Microcystis* abundance well in two similar deep water stations in Lake Mendota, but not in any of the other lakes or stations sampled. In neighboring Lake Monona *Microcystis* was correlated with SRP, but not stratification. However, this was likely due to the fact that 2 of 3 Lake Monona stations were shallow. In other studies, temporal variability in *Microcystis* has been correlated with both stratification and SRP, but usually only in a single lake or multiple lakes with poor temporal resolution. Hence, heterogeneity in cyanobacterial ecology, genetics, and physiology within and between lakes is likely even greater than currently recognized. We should note that only a subset of all possible environmental variables were measured in this study.

We showed that there is significant within lake spatial heterogeneity, particularly with respect to cyanobacterial genetic structure and overall abundance. For example, trends in phycocyanin concentration were not correlative between any two locations and except for Lake Mendota, the relative abundance of individual taxa at locations within the same lake followed contrasting trajectories through time (Figure 4). These data suggests that conclusions based on single sampling points in a lake are unlikely to be directly transferrable to other locations within a lake. Furthermore the data support further research focused on improving three- dimensional coupled hydrodynamic-ecosystem modeling approaches (e.g. 41) in order to capture this heterogeneity.

We observed that CCC was significantly segregated between stations according to depth of the water column. Accordingly, of all environmental variables measured, ∆T was most strongly correlated with spatial variability in CCC (Figure 2, Table 2). Water column stability is thought to favor growth of some cyanobacteria and previous studies have noted differences in CCC between deep and shallow lakes [42]. For example, the Oscillatoriales are often observed to dominate shallow systems [43,44]. However, this is clearly not always the case since Chroococcales such as *Microcystis* have been observed to dominate many shallow lakes including Lake Taihu, a well-studied shallow eutrophic lake in Eastern China [45]. In this study, *Microcystis* genera were more abundant in shallow locations, especially Lake Wingra, but also emerged later in the Lake Mendota deep locations when stratification was strong. In a study by Brock et al. (1985) in Lake Mendota, *Microcystis* were more often observed in deep sediment traps than *Aphanizomenon* . Thus, strong vertical stability may be important in order for *Microcystis* maintain significant biomass in the photic zone of deep locations, whereas in shallow locations, the water column in thoroughly mixed with light penetrating throughout. This may be a result of poorer buoyancy control compared to *Aphanizomenon* , or perhaps *Microcystis* are better adapted to high light conditions in these lakes.

The affect of depth of the water column on cyanobacterial ecology is well studied and may be explained by light and nutrient availability regulated by water column stratification [46,47]. In this study, PZD extended through nearly the entire water column on most sampling days at shallow locations. The PZD was important for temporal variability in CCC (Table 3), but not spatial variability between stations (Table 2). Aside from **Δ**T, dissolved inorganic nutrients were also significantly correlated with spatial variability in overall CCC. All shallow stations had slightly lower mean concentrations of standing stock SRP, which may have been due to co-occurrence of macrophytes in these areas. Thus, it appears that nutrient availability and eukaryotic competition may play a larger role in structuring CCC between shallow and deep locations within these lakes than does PZD. This agrees with the average higher coefficient of variation in nutrient levels (0.9-1.6) compared to the PZD (0.4-0.6) at all locations.

Temporal variations in CCC were most strongly correlated with PZD and/or SRP, and to a lesser extent with nitrate, water temperature, and DO. In 2008 when this study was conducted, spring rainfall was abnormally high resulting in significant flooding across the region in June. Accordingly, dissolved inorganic nutrients were highest in June. This came during a period of increasing stratification and well after the spring mixing event that begins during ice-off (March or April). The strong pulse of nutrients delivered during these rain events could have disrupted any seasonal trend in CCC, which was not observed in our dataset. Rather the influx of inorganic nutrients, particularly SRP, may have structured CCC during this year. Cyanobacterial blooms of *Aphanizomenon* and, to a lesser extent, *Microcystis* quickly increased after the June rain events as nutrients and PZD declined while water temperature increased (Table 4, Figure S1). Thus, it is likely that June rainfall events and associated nutrient influx played a large role in shaping the temporal variation observed in bloom formation and CCC during 2008. As climate change is expected to induce extreme weather events, the 2008 flooding may be representative of future years.

We observed a strong anti-correlation between *Aphanizomenon* and *Microcystis* . This anti-correlation is primarily due to the occurrence of *Aphanizomenon* blooms early in summer while *Microcystis* had peak abundances later in summer into early autumn at most locations (Figure S1). In addition, at some stations (e.g. MEDH, KEBE) the population cycled between *Microcystis* and *Aphanizomenon* dominance two or three times throughout the sampling period. Our data do not allow for a definitive explanation of this phenomenon. However, there are several possibilities.

The N_2_- fixing cyanobacteria such as *Aphanizomenon* are expected to out compete other non-N_2_- fixing cyanobacteria when N:P ratios decline below some threshold, which could be higher than the Redfield ratio [48]. At most stations, nutrients were just beginning to decline from peak concentrations in early June when *Aphanizomenon* blooms were observed and the N+N:SRP ratio was low or declining. It is possible this decline was significant enough to trigger N_2_- fixation giving some advantage to early blooms of *Aphanizomenon* and not *Microcystis* . A recent study by Beversdorf et al. in Lake Mendota suggests that N_2_- fixation by *Aphanizomenon* during nutrient draw down early in the summer provides new N input supporting toxic *Microcystis* blooms later in summer [20]. As this new N is consumed, N_2_- fixing *Aphanizomenon* are favored thus explaining cycling between *Aphanizomenon* and *Microcystis* .

On the other hand, early blooms of *Aphanizomenon* may be explained by a slightly lower optimal growth temperature for *Aphanizomenon* compared to that of *Microcystis* . At the start of our study in June, water temperatures were at 15 °C, increased to 27 °C in early July and then reached a maximum of 30 °C at all stations between mid- July and early August. Most, but not all *Aphanizomenon* blooms occurred before or at the time max temperatures were reached, while many of the *Microcystis* blooms occurred after max temperatures were reached. A literature review by Robarts and Zohary indicate that growth of *Microcystis* stops below 15 °C with optimal growth temperatures of 27.5-32 °C. In contrast, the optimal growth temperature for four strains of *Aphanizomenon* was 15-28 °C [49]. This suggests that *Aphanizomenon* may have the ability to outgrow *Microcystis* at lower temperatures, such as earlier in the summer of temperature lakes. This agrees with our observations of earlier blooms of *Aphanizomenon* . However, we did not examine optimal growth temperatures of the *Aphanizomenon* taxa in our lakes and other drivers may be more important.

Another possibility is that anti-predation characteristics specifically possessed by *Aphanizomenon* in these lakes may be important for establishing conditions that allow cyanobacteria to dominate, transitioning out of the “clear water” phase when zooplankton are abundant. Both *Microcystis* and *Aphanizomenon* display anti-predation phenotypes including colony formation, toxin production, and overall prey avoidance [50,51]. It is possible that a combination of lower optimal growth temperature, anti- predation characteristics, and N_2_- fixation provide a competitive advantage for *Aphanizomenon* earlier in the summer during the transition out of the zooplankton dominated clear- water phase. This hypothesis requires more investigation, but should be fruitful since much of the cyanobacterial bloom literature has focused on *Microcystis* blooms.

As we look to forecast the effects of management decisions, climate and land use change on the occurrence and frequency of algal bloom events, it is necessary to characterize taxa responsible for producing this biomass. We can draw some very salient parallels to the infectious disease literature, which is right now focused on the necessity of differentiating bacterial pathogens based on genetic markers rather than phenotypic morphological markers [51]. Should we treat cyanobacteria any differently? This study shows that not all taxa of the bloom- forming genera in lakes (e.g. *Microcystis* or *Aphanizomenon* ) are responsible for producing the bulk of biomass in lakes. In fact, only one of three *Microcystis* OTUs was responsible for most bloom events indicating that these “species” differ greatly in their ability to dominate the water column. Therefore, the identification of taxa that specifically contribute to blooms at a genetic level is likely to provide a better understanding of CCC and overall cyanobacterial bloom ecology.

### Conclusions

This study provided the most comprehensive analysis to date of spatial and temporal variability in CCC across multiple lakes and within-lake stations using a culture- independent molecular approach. The CCC across all stations was most strongly segregated by depth of the sampling location and was correlated most strongly with thermal stratification and nutrients. Spatial variability in CCC and temporal trends in taxa abundance were rarely correlative between sampling stations in the same lake indicating significant heterogeneity in the x-y dimensions. More than 90 taxa were identified, yet a single *Microcystis* , and two different *Aphanizomenon* taxa were the dominant cyanobacteria detected during all bloom events at all stations including multiple stations in the same lake. The *Microcystis* and *Aphanizomenon* taxa were significantly anti-correlated with each other at most stations and *Aphanizomenon* was more abundant when nutrients were higher suggesting interesting interactions between these genera potentially driven by N_2_-fixation or other phenotypes (e.g. optimal growth temperature, anti- predation characteristics). Overall, this study shows that temporal trends in biomass and individual taxa are heterogeneous between lakes and locations in the same lake. Furthermore, the molecular approach shows that just a few individual genotypes were responsible for most bloom events.

## Supporting Information

Table S1 **Results of pair-wise comparisons of cyanobacterial community composition between locations in the same lake using ANOSIM.**
(DOCX)

Table S2 **Significant (P<0.05) correlations (Pearson R) between the major bloom forming *Aphanizomenon* , or *Microcystis* taxa and environmental variables.**
(DOCX)

Figure S1 **Trends in biological, physical, and chemical variables across all lakes and locations.** For each location the top graph represents the sum of the relative abundance of all *Microcystis* taxa compared to that of *Aphanizomenon* taxa. Arrows indicate peak bloom dates based on phycocyanin. The second graph shows changes in phycocyanin and water temperature in the photic zone. The photic zone depth (PZD) was estimated from secchi disk depth, which is shown in the third graph along with the difference in water temperature from surface to lake bottom as a measure of water column stratification (**Δ**T). The fourth graph shows trends in nutrients (TP = total phosphorus, SRP = soluble reactive phosphorus). and the nitrate+nitrite to SRP ratio (N+N:SRP). Nitrate and nitrite were below detection limits in Lake Wingra.
(TIF)

Figure S2 **Cyanobacterial surface scums on Lake Kegonsa, WI, August 2008.**
(TIF)

Figure S3 **Differences in mean relative abundance of identified taxa between deep and shallow locations.** Chr352, Mcy215, and Unmatched taxa are significant differences.
(TIF)
