# Supplementary material for: Spatiotemporal Molecular Analysis of Cyanobacteria Blooms Reveals Microcystis - Aphanizomenon Interactions
Source: PLoS One. 2013 Sep 27;8(9):e74933. doi: 10.1371/journal.pone.0074933 (PMC3785500; doi:10.1371/journal.pone.0074933)
Supplement: Table S1 — Results of pair-wise comparisons of cyanobacterial community composition between locations in the same lake using ANOSIM. (DOCX) [file pone.0074933.s001.docx]

| **Locations^a^** | **R** | **p** |
| --- | --- | --- |
| MEDH (D), MEPP (S) | 0.196 | 0.003 |
| MEGA (D), MEPP (S) | 0.035 | 0.13 |
| MEDH (D), MEGA (S) | 0.379 | 0.001 |
| MODH (D), MOBE (S) | 0.301 | 0.002 |
| MODH (D), MOMB (S) | 0.259 | 0.001 |
| MOMB (S), MOBE (S) | 0.054 | 0.082 |
| KEDH (D), KEYA (S) | 0.271 | 0.001 |
| KEDH (D), KEBE (S) | 0.023 | 0.212 |
| KEBE (S), KEYA (S) | 0.111 | 0.023 |
| WIAR (S), WIBE (S) | 0.016 | 0.242 |
| WIAR (S), WIDH (S) | 0.019 | 0.311 |
| WIBE (S), WIDH (S) | 0.067 | 0.118 |

**Table S1.** Results of pair-wise comparisons of CCC between locations in the same lake using ANOSIM

^a^Deep locations are marked with a “D” and shallow locations with an “S.”
