# Supplementary material for: Spatiotemporal Molecular Analysis of Cyanobacteria Blooms Reveals Microcystis - Aphanizomenon Interactions
Source: PLoS One. 2013 Sep 27;8(9):e74933. doi: 10.1371/journal.pone.0074933 (PMC3785500; doi:10.1371/journal.pone.0074933)
Supplement: Table S2 — Significant (P<0.05) correlations (Pearson R) between the major bloom forming Aphanizomenon , or Microcystis taxa and environmental variables. (DOCX) [file pone.0074933.s002.docx]

**Table S2.** Significant (P<0.05) correlations (Pearson R) between the major bloom forming *Aphanizomenon,* or *Microcystis* taxa and environmental variables.

| **Site** | **Taxa** | **TP** | **SRP** | **Nitrate** | **Nitrite** | **deltaT** | **DO** | **PZD** | **Temp** |
| --- | --- | --- | --- | --- | --- | --- | --- | --- | --- |
| MEDH | Aph680 |  |  |  |  | 0.71 | 0.65 |  | 0.68 |
|  | Aph700 |  | 0.63 | 0.5 |  |  |  |  |  |
|  | Aph |  |  |  |  | 0.60 | 0.56 |  | 0.55 |
|  | Mcy215 |  |  |  |  | -0.53 | -0.61 |  | -0.50 |
|  | Mcy |  | -0.51 |  |  | -0.48 | -0.55 |  |  |
| MEGA | Aph680 |  |  | -0.51 |  |  | 0.61 | -0.55 | 0.62 |
|  | Aph700 |  | 0.71 |  |  |  | 0.51 |  |  |
|  | Aph |  | 0.50 |  |  | 0.52 | 0.68 |  | 0.53 |
|  | Mcy215 |  |  |  |  |  |  |  |  |
|  | Mcy |  |  |  |  | -0.52 | -0.66 |  | -0.63 |
| MEPP | Aph680 |  |  |  |  |  |  | -0.53 |  |
|  | Aph700 |  | 0.84 | 0.62 | 0.65 |  |  | 0.68 |  |
|  | Aph |  | 0.70 |  |  |  | 0.59 |  |  |
|  | Mcy215 |  |  |  |  |  |  |  |  |
|  | Mcy |  | -0.69 |  |  |  | -0.69 |  |  |
| MODH | Aph680 |  | 0.58 | 0.57 |  |  | 0.62 | 0.59 |  |
|  | Aph700 |  |  | 0.56 |  |  |  |  |  |
|  | Aph |  | 0.63 | 0.80 |  |  |  | 0.61 |  |
|  | Mcy215 |  |  |  |  |  |  |  |  |
|  | Mcy |  | -0.55 | -0.73 | -0.49 |  | -0.53 |  |  |
| MOMB | Aph680 |  |  |  |  | 0.55 | -0.61 |  | 0.53 |
|  | Aph700 |  |  | -0.58 |  |  |  |  |  |
|  | Aph |  |  |  |  |  | -0.57 |  |  |
|  | Mcy215 | 0.73 |  | 0.53 |  |  |  |  |  |
|  | Mcy | 0.53 |  | 0.57 |  |  |  |  |  |
| MOBE | Aph680 |  |  |  |  | 0.58 |  |  |  |
|  | Aph700 |  |  |  |  |  |  | 0.52 |  |
|  | Aph |  |  |  |  |  |  | 0.47 |  |
|  | Mcy215 |  |  |  |  |  |  |  |  |
|  | Mcy |  |  |  |  |  |  |  |  |
| KEDH | Aph680 |  |  |  |  |  |  |  |  |
|  | Aph700 | -0.54 |  |  |  |  |  | 0.66 |  |
|  | Aph |  |  |  |  |  |  |  |  |
|  | Mcy215 |  |  |  |  |  |  | 0.52 | -0.60 |
|  | Mcy |  |  |  |  |  |  |  | 0.48 |
| KEYA | Aph680 |  |  |  | -0.55 |  |  |  |  |
|  | Aph700 |  |  |  |  |  |  |  |  |
|  | Aph |  |  |  |  |  |  |  |  |
|  | Mcy215 |  |  |  |  |  |  |  | -0.51 |
|  | Mcy |  |  |  |  |  |  |  |  |
| KEBE | Aph680 |  | 0.66 |  |  |  |  | 0.60 | 0.76 |
|  | Aph700 |  |  |  |  |  | 0.52 |  |  |
|  | Aph |  | 0.72 |  |  |  |  | 0.59 | 0.68 |
|  | Mcy215 |  | -0.62 |  |  |  |  |  |  |
|  | Mcy |  | -0.54 |  |  |  |  |  | -0.53 |
