# Supplementary figures and images for: Spatiotemporal Molecular Analysis of Cyanobacteria Blooms Reveals Microcystis - Aphanizomenon Interactions
Source: PLoS One. 2013 Sep 27;8(9):e74933. doi: 10.1371/journal.pone.0074933 (PMC3785500; doi:10.1371/journal.pone.0074933)

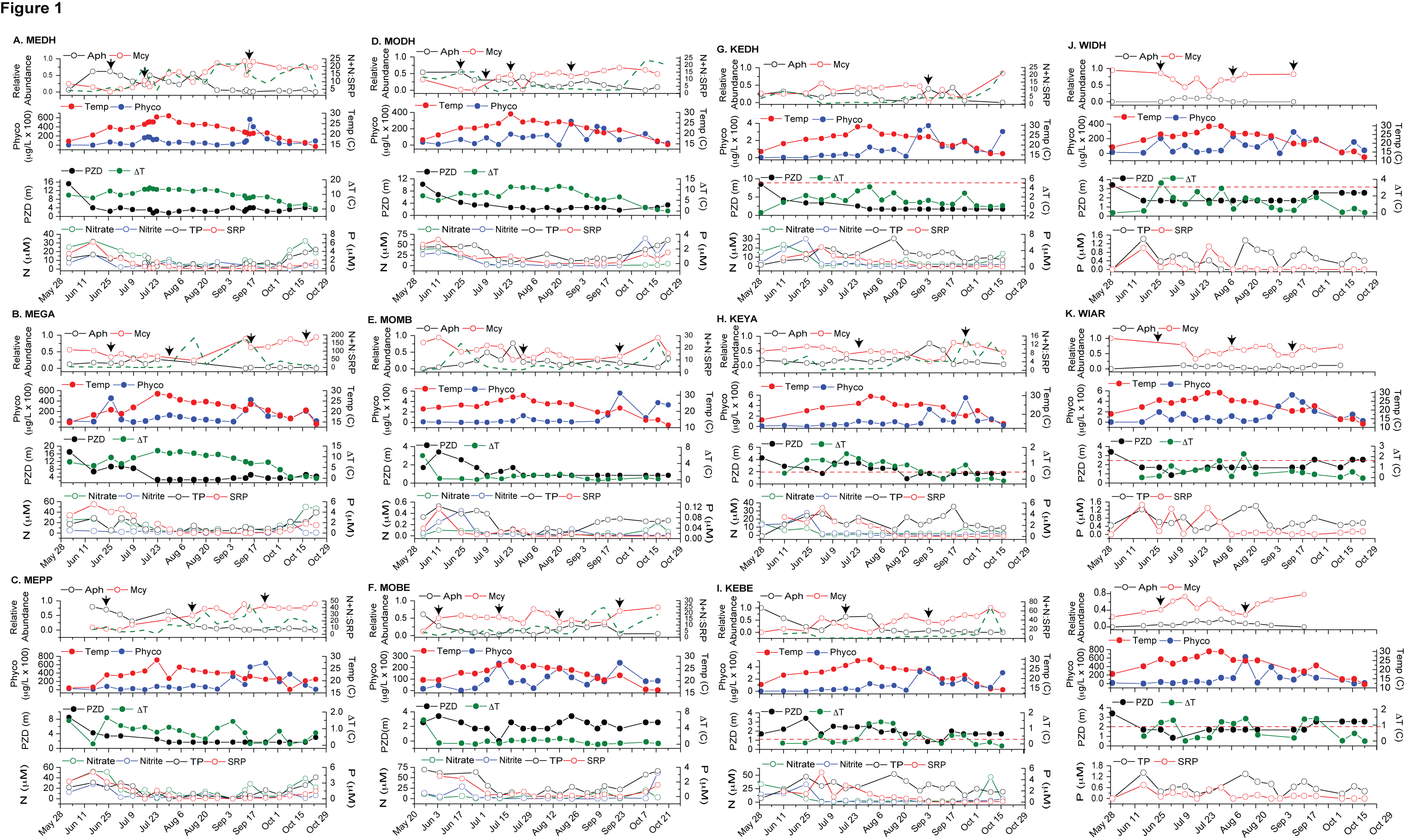

Supplement: Figure S1 — Trends in biological, physical, and chemical variables across all lakes and locations. For each location the top graph represents the sum of the relative abundance of all Microcystis taxa compared to that of Aphanizomenon taxa. Arrows indicate peak bloom dates based on phycocyanin. The second graph shows changes in phycocyanin and water temperature in the photic zone. The photic zone depth (PZD) was estimated from secchi disk depth, which is shown in the third graph along with the difference in water temperature from surface to lake bottom as a measure of water column stratification (ΔT). The fourth graph shows trends in nutrients (TP = total phosphorus, SRP = soluble reactive phosphorus). and the nitrate+nitrite to SRP ratio (N+N:SRP). Nitrate and nitrite were below detection limits in Lake Wingra. (TIF) [file pone.0074933.s003.tif]

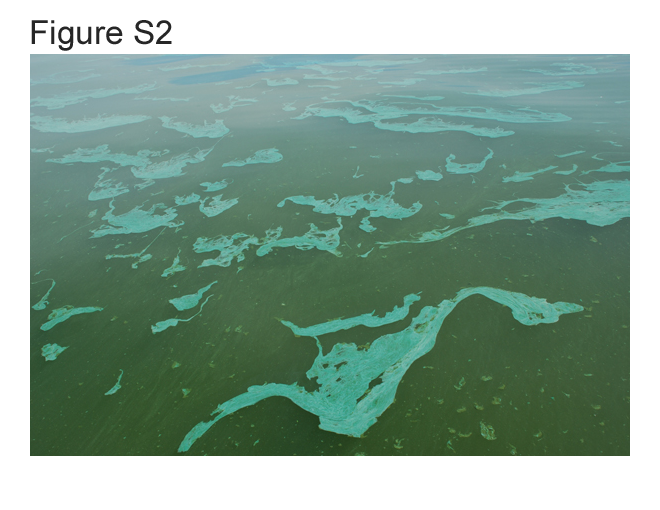

Supplement: Figure S2 — Cyanobacterial surface scums on Lake Kegonsa, WI, August 2008. (TIF) [file pone.0074933.s004.tif]

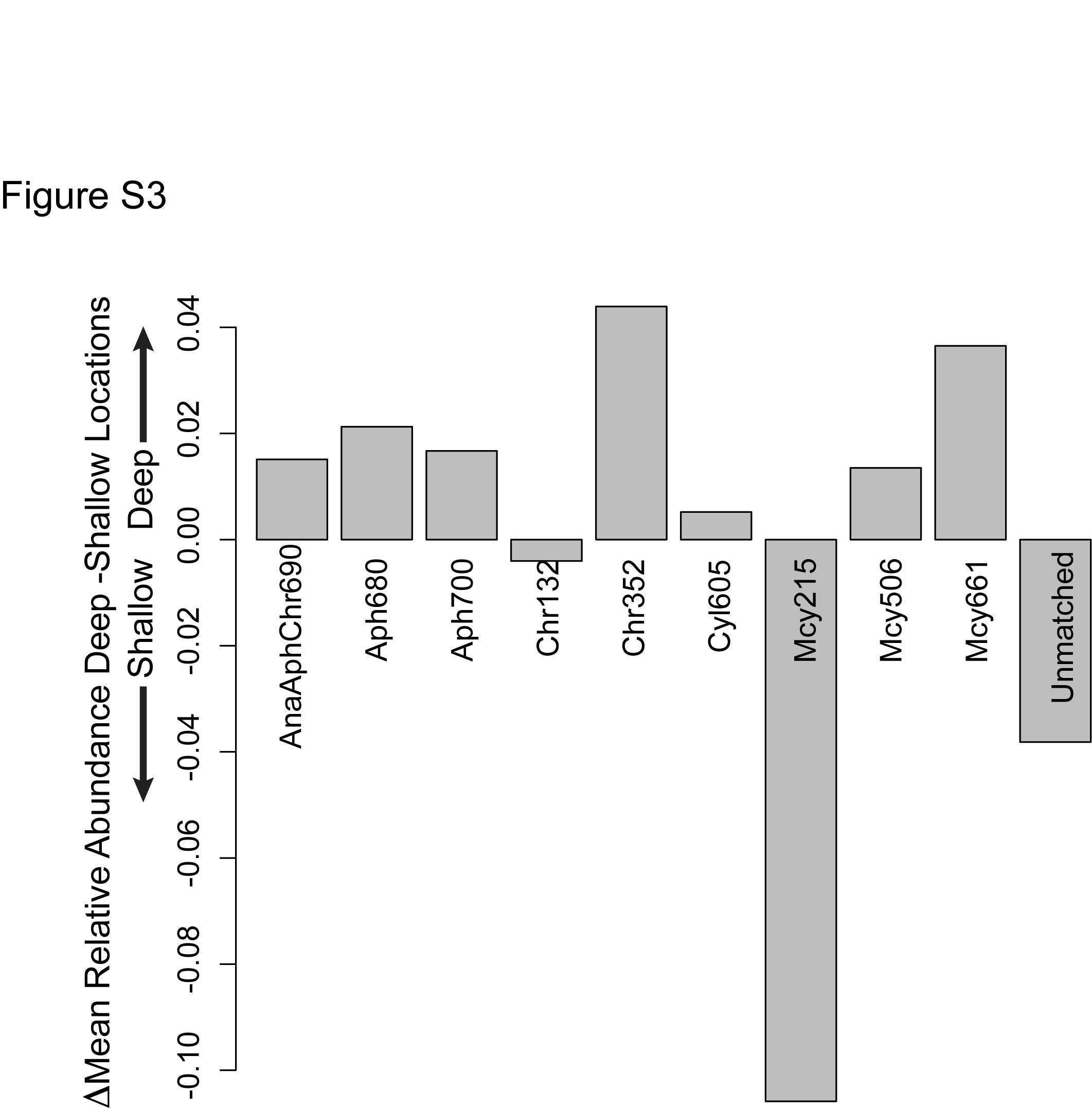

Supplement: Figure S3 — Differences in mean relative abundance of identified taxa between deep and shallow locations. Chr352, Mcy215, and Unmatched taxa are significant differences. (TIF) [file pone.0074933.s005.tif]
